# Self-Assembled Polymeric Micelles Based on Hyaluronic Acid-*g*-Poly(d,l-lactide-co-glycolide) Copolymer for Tumor Targeting

**DOI:** 10.3390/ijms150916057

**Published:** 2014-09-11

**Authors:** Gyung Mo Son, Hyun Yul Kim, Je Ho Ryu, Chong Woo Chu, Dae Hwan Kang, Su Bum Park, Young-IL Jeong

**Affiliations:** 1 Department of Surgery, National Research & Development Center for Hepatobiliary Cancer, Pusan National University Yangsan Hospital, Gyeongnam 626-770, Korea; E-Mails: gyungmoson@outlook.kr (G.M.S.); hyunyulkim@outlook.kr (H.Y.K.); jehoryu@outlook.kr (J.H.R.); chongwoochu@outlook.kr (C.W.C.); 2 Biomedical Research Institute, Pusan National University Hospital, Pusan 602-739, Korea; E-Mail: nanomed@naver.com; 3 Department of Gastroenterology, Pusan National University Hospital, Pusan 602-739, Korea; 4 Department of Internal Medicine, Pusan National University Yangsan Hospital, Gyeongnam 626-770, Korea

**Keywords:** hyaluronic acid, polymeric micelles, HepG2 hepatoma cells, CD44 receptor

## Abstract

Graft copolymer composed hyaluronic acid (HA) and poly(d,l-lactide-co-glycolide) (PLGA) (HAgLG) was synthesized for antitumor targeting via CD44 receptor of tumor cells. The carboxylic end of PLGA was conjugated with hexamethylenediamine (HMDA) to have amine end group in the end of chain (PLGA-amine). PLGA-amine was coupled with carboxylic acid of HA. Self-assembled polymeric micelles of HAgLG have spherical morphologies and their sizes were around 50–200 nm. Doxorubicin (DOX)-incorporated polymeric micelles were prepared by dialysis procedure. DOX was released over 4 days and its release rate was accelerated by the tumoric enzyme hyaluronidase. To assess targetability of polymeric micelles, CD44-positive HepG2 cells were employed treated with fluorescein isothiocyanate (FITC)-labeled polymeric micelles. HepG2 cells strongly expressed green fluorescence at the cell membrane and cytosol. However, internalization of polymeric micelles were significantly decreased when free HA was pretreated to block the CD44 receptor. Furthermore, the CD44-specific anticancer activity of HAgLG polymeric micelles was confirmed using CD44-negative CT26 cells and CD44-positive HepG2 cells. These results indicated that polymeric micelles of HaLG polymeric micelles have targetability against CD44 receptor of tumor cells. We suggest HAgLG polymeric micelles as a promising candidate for specific drug targeting.

## 1. Introduction

Self-assembling properties of amphiphilic macromolecules have been extensively investigated for drug targeting because they have drug carrying potential to a specific site of the human body or cells [1,2,3]. Especially, polymeric micelles or core-shell type nanoparticles have unique structural properties,* i.e.*, polymeric micelles have a hydrophobic inner-core and hydrated outer-shell structure. Inner-core of the polymeric micelles can act as a drug incorporation domain and hydrated outer-shell is normally helpful to avoid unwanted uptake by reticuloendothelial system (RES) [1]. In most reports, however, hydrated outer-shell of polymeric micelles have been designed with poly(ethylene glycol) (PEG),* i.e.*,PEG has no specific functionality even though it is helpful to increase the blood circulation times of vehicles and to avoid non-specific binding with normal cells.

HA is a component of the extracellular matrix and has a key role in cell adhesion, growth, and migration [4,5]. HA is also responsible in cell motility, inflammation, wound healing, and cancer invasion/metastasis [6,7,8]. Furthermore, HA is known to have specificity against CD44 receptor of tumor cells [4,5]. Especially, HA receptors such as CD44 and RHAMM are over-expressed at malignant cells having high mobility and invasive capacity [9,10]. From this point of view, HA has been extensively used as a targeting molecule for CD44 receptor of tumor cells or invasive tumor cells [4,5,11,12,13]. For example, Luo and Prestwich reported that hydrophobic anticancer drug was conjugated to HA for cancer targeting via CD44 receptor [5]. Jeong* et al.* also reported that cisplatin was incorporated into HA nanoparticles via ion complex formation and then release rate of cisplatin can be accelerated by the tumoric enzyme hyaluronidase [4]. Since HA is a biocompatible molecule, HA is excellent for site specific targeting of anticancer drugs to tumor cells and for modification of medical polymers [14,15].

In this study, we synthesized graft copolymer composed of hyaluronic acid (HA) and poly(d,l-lactide-co-glycolide) (PLGA) to fabricate polymeric micelles for active drug targeting of CD44 receptor. Since HA is an aqueous soluble molecule, HA can form corona of the polymeric micelles and PLGA can form inner-core as a drug incorporation site. Physicochemical properties of HagLG polymeric micelles were studied and their drug targeting potential was investigated against CD44 receptor of cancer cells* in vitro*.

## 2. Results and Discussion

### 2.1. Characterization Hyaluronic Acid (HA) and Poly(d,l-lactide-co-glycolide) (PLGA) (HAgLG) Copolymer and HAgLG Polymeric Micelles

**Figure 1:**
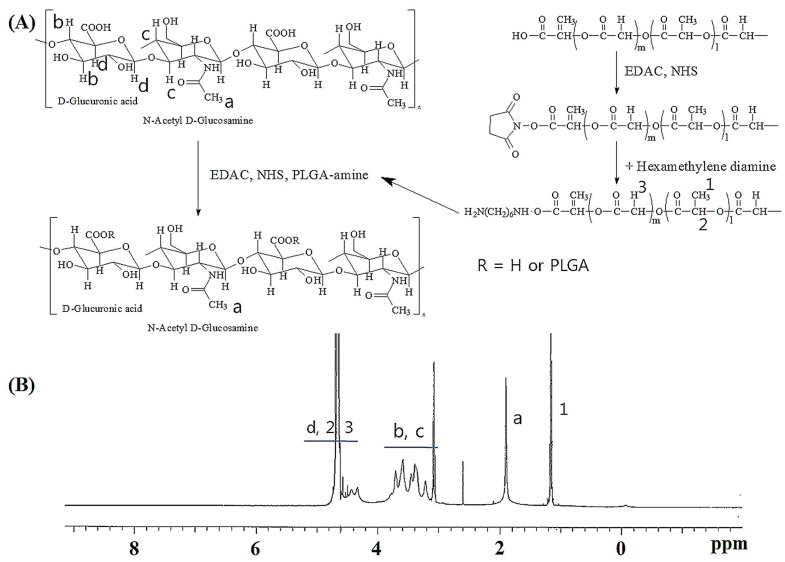
(**A**) Synthesis scheme and (**B**) ^1^H NMR spectra of Hyaluronic Acid (HA) and Poly(d,l-lactide-co-glycolide) (PLGA) copolymer (HAgLG).

To synthesize HA and PLGA (HAgLG) copolymer, carboxylic group of PLGA was aminated with hexamethylene diamine (HMDA) and then aminated PLGA was conjugated with carboxylic acid group of HA with the aid of *N*-(3-dimethylaminopropyl)-*N*'-ethylcarbodiimide hydrochloride (EDAC) and *N*-hydroxysuccinimide (NHS) (Figure 1). At ^1^H NMR spectra, methyl group of PLGA and acetyl group of HA was confirmed at 1.4 and 1.9 ppm, respectively. Since the acetyl protons in HA and methylene protons of PLGA were appeared at 1.9 and 1.2 ppm, respectively, the substitution degree (DS) of PLGA in the HA backbone was estimated by comparison of these peaks (Figure 1). The various DS of PLGA in the HAgLG copolymer was synthesized with different feeding amounts of aminated PLGA as shown in Table 1. The DS of PLGA* versus* 100 units of disaccharides (disaccharide unit composed of d-glucuronic acid and *N*-acetyl-d-glucosamine in the HA backbone) was evaluated.

**Table 1:** Characterization of HAgLG polymeric micelles.

HAgLG Copolymer	DS of PLGA *	Particle Size (nm)
HAgLG-1	4.9	49.5 ± 10.8
HAgLG-2	9.4	81.3 ± 18.1
HAgLG-3	18.6	102.6 ± 23.6

* DS of PLGA was estimated *versus* 100 units of disaccharide of HA. HA: hyaluronic acid; DS: substitution degree; PLGA: poly(d,l-lactide-co-glycolide); HAgLG: HA and PLGA copolymer.

**Figure 2:**
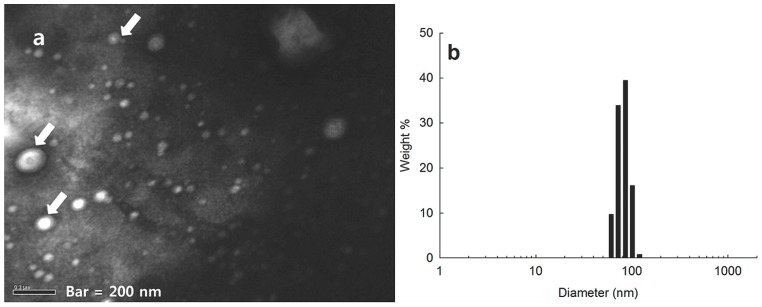
TEM photograph (**a**) and typical particle size (**b**) of HAgLG polymeric micelles. Polymeric micelles were stained with phosphotungstic acid (0.1% (*w*/*v*). Arrows indicated that spherical particles have core-shell type micellar structure.

Polymeric micelles of HAgLG graft copolymer was fabricated by dialysis procedure. To confirm formation of polymeric micelles, particle size and morphology of HAgLG were confirmed with dynamic light scattering (DLS) and transmission electron microscope (TEM) as shown in Figure 2. Polymeric micelles of HAgLG have small particle sizes less than 100 nm and are spherical in shape. Their average particle size was 81 nm. These results indicated that HAgLG graft copolymer can form spherical particles with small diameters.

**Figure 3:**
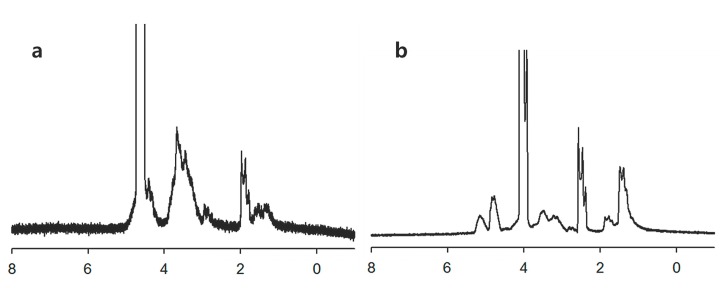
^1^H NMR spectra of core-shell type nanoparticles of HAgLG copolymer in D_2_O (**a**) and DMSO (**b**).

Since amphiphilic macromolecules have self-assembling properties in aqueous environment, their structural properties were investigated with ^1^H NMR spectra using D_2_O (Figure 3). Water-soluble HA backbone should form the outer shell of polymeric micelles while PLGA form the inner-core. As expected, peaks of HA in the polymeric micelles were only confirmed at D_2_O while PLGA peaks disappeared. However, peaks both of HA and PLGA appeared at D_2_O/DMSO mixtures, indicating that HAgLG formed polymeric micelles at aqueous solution and HA formed the outer shell of the polymeric micelles.

To assess characteristics of HAgLG polymeric micelles as an anticancer drug targeting system, doxorubicin (DOX) was incorporated into the HAgLG polymeric micelles. As summarized in Table 2, DOX-incorporated polymeric micelles showed increased average particle sizes compared to empty micelles, * i.e.*, particle sizes of DOX-incorporated polymeric micelles were increased to 180 nm and higher drug feeding ratio induced increased particle sizes. The higher LG contents induced higher drug loading contents and particle size. Furthermore, lyophilized polymeric micelles were reconstituted to study lyophilization effect on the characteristics of polymeric micelles. As shown in Table 1, size of polymeric micelles after lyophilization procedure was not significantly changed.

**Table 2:** Characterization of doxorubicin (DOX)-incorporated HAgLG polymeric micelles.

HAgLG Copolymer	Drug/Polymer Weight (mg/mg)	Drug Contents (%, *w*/*w*)		Particle Size (nm)		
		Theoretical	Experimental	Before Lyophilization	After Lyophilization	Statistical Analysis *
HAgLG-1	5/40	11.1	5.4	89.6 ± 19.6	93.0 ± 21.0	>0.001
HAgLG-2	5/40	11.1	6.2	118.4 ± 23.1	124.3 ± 28.2	>0.01
	10/40	20	10.8	153.8 ± 31.2	170.6 ± 34.6	>0.01
HAgLG-3	5/40	11.1	8.3	180.9 ± 36.8	193.8 ± 40.8	>0.05

* Statistical analysis was adapted with three or four times measurement of particle size after reconstitution of lyophilized nanoparticles into deionized water.

**Figure 4:**
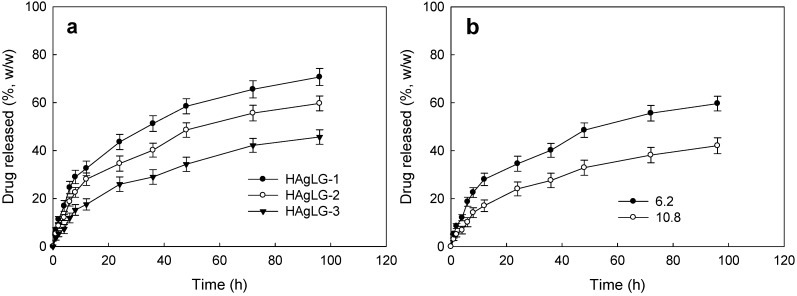
DOX release from HAgLG polymeric micelles. The effect of PLGA contents in the copolymer (**a**); drug contents in the polymeric micelles (**b**).

**Figure 5:**
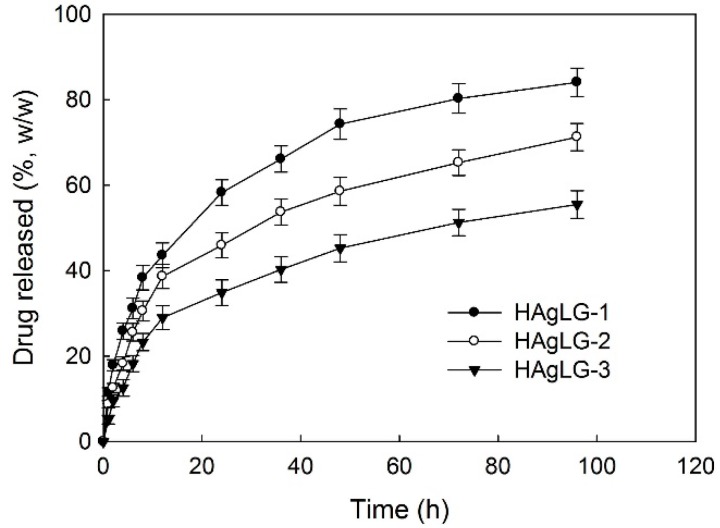
The effect of hyaluronidase addition on the drug release. To study the effect of hyaluronidase, hyaluronidase was added to release experiment of DOX-incorporated HAgLG polymeric micelles (5 mg of DOX-incorporated HAgLG polymeric micelles in dialysis tube with 5 mL PBS and 100 unit HAse).

DOX release was assessed *in vitro* as shown in Figure 4 and Figure 5. Higher LG contents in the graft copolymer showed delayed release properties compared to copolymers having lower LG contents as shown in Figure 4a. Furthermore, higher drug loading contents showed slower drug release kinetics. These phenomena might be due to the fact that hydrophobic drugs such as DOX were aggregated in the core of the polymeric micelles at higher levels, while hydrophobic drugs at lower levels existed as molecular dispersions in the core of the polymeric micelles, and aggregated drugs released slower than molecular dispersions [2,3].

Generally, cancer cells demonstrating invasiveness and aggressiveness frequently secreted hyaluronidase to degrade extracellular matrix and to invade surrounding tissues [4]. Figure 5 showed the effect of hyaluronidase addition on the release of DOX from polymeric micelles. As shown in Figure 5, interestingly, hyaluronidase addition in the release test of DOX-incorporated polymeric micelles resulted in accelerated drug release kinetics compared to that with an absence of hyaluronidase. These results were due to the fact that hyaluronidase degrades the hyaluronic acid segment of polymeric micelles and then DOX release rate was accelerated compared to that with an absence of hyaluronidase. These results indicated that the tumoric enzyme hyaluronidase specifically modified drug release kinetics from polymeric micelles and DOX-incorporated HAgLG polymeric micelles responded to hyaluronidase secretion of cancer cells.

### 2.2. Receptor-Mediated Targeting of HAgLG Polymeric Micelles

**Figure 6:**
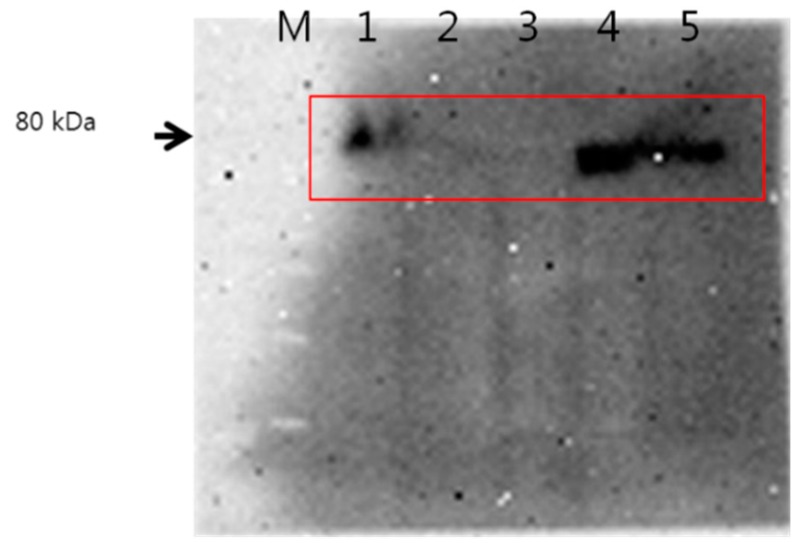
Western blot analysis of tumor cells. M: Marker; 1: SK Hep01; 2: CT26; 3: empty; 4: KB; 5: HepG2. Rectangle area: CD44.

Prior to assess targetability of HAgLG polymeric micelles against CD44 receptor of tumor cells, CD44 receptor expression at various cell lines was studied using western blot analysis as shown in Figure 6. As shown in Figure 6, HepG2 and KB cells strongly expressed CD44 protein among all of the cell lines. Therefore, HepG2 cells were used to study receptor-mediated delivery of HAgLG polymeric micelles.

**Figure 7:**
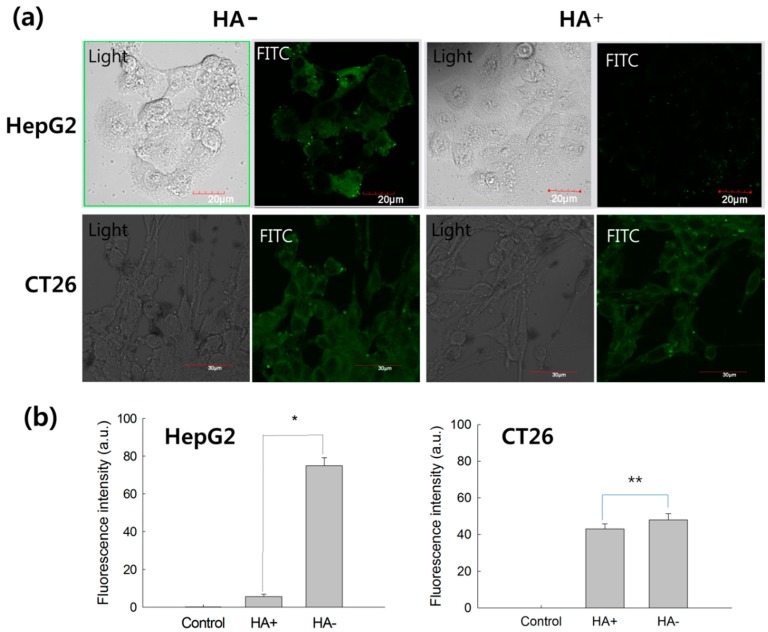
(**a**) Fluorescence images of HepG2 cells and CT26 cells. Cancer cells were exposed to fluorescein isothiocyanate (FITC)-labelled HAgLG-2 polymeric micelles (10 µg/mL) for 1 h. Fluorescence images of cells were observed with CLSM. To confirm receptor-mediated endocytosis of nanoparticles, CD44 receptor was blocked with (HA+) or without (HA−) 10 times higher amount of free HA; (**b**) Flow cytometric analysis of FITC-labelled HAgLG-2 polymeric micelle-treated cells with or without blocking of CD44 receptor. The experiment was triplicated and expressed as average ± S.D. * *p* < 0.001; ** *p* < 0.01.

To assess targetability of HAgLG polymeric micelles, fluorescein isothiocyanate (FITC)-labeled polymeric micelles were treated to HepG2 cells and CT26 cells. Cells were observed with fluorescence microscopy as shown in Figure 7a. To study receptor-mediated delivery of HAgLG polymeric micelles, CD44 receptor of HepG2 cells were blocked by pre-treatment with 10 equal quantities of free HA 1 h before polymeric micelles’ treatment. Results showed that HepG2 cells strongly expressed green fluorescence at cell surface and cytosol when cells were not blocked with HA. Fluorescence intensity of HepG2 cells was significantly decreased when CD44 receptor was blocked with free HA (Figure 7a,b). However, fluorescence intensity of CT26 cells was not significantly changed by blocking of CD44 receptor (Figure 7a,b). These results indicated that HAgLG polymeric micelles can be delivered through CD44 receptor of HepG2 cells. Furthermore, to quantitatively investigate the receptor-mediated endocytosis of HAgLG polymeric micelles, cells were exposed to FITC-labelled HAgLG polymeric micelles and then analyzed by flow cytometry as shown in Figure 7b. When HepG2 cells were exposed to HAgLG polymeric micelles, fluorescence intensity was significantly increased. On the other hand, when CD44 receptor of tumor cells was blocked with free HA (HA+ in Figure 7b), fluorescence intensity was obviously decreased. Fluorescence intensity of CT26 cells was not significantly decreased by pretreatment of HA (Figure 7b). These results indicated that HAgLG polymeric micelles entered HepG2 cells via CD44 receptor-mediated endocytosis.

**Figure 8:**
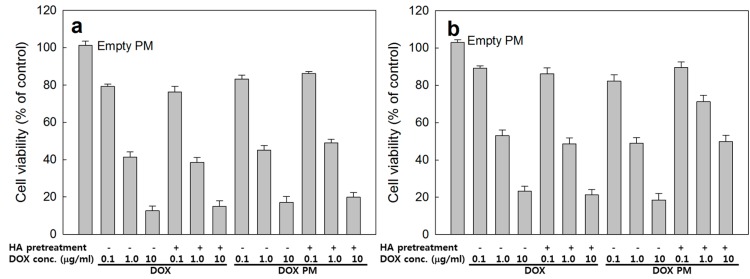
Anticancer activity of DOX-incorporated HAgLG-2 polymeric micelles (Drug contents: 10.8% (*w*/*w*)) against CT26 cells (**a**) and HepG2 cells (**b**). Cells were pretreated with HA (10 times higher amount of PM content) 1 h before drug treatment to block CD44 receptor. Empty PM: empty polymeric micelles (HAgLG-2); DOX: doxorubicin HCl; DOX PM: DOX-incorporated polymeric micelles (HAgLG-2 micelles with 10.8% (*w*/*w*) drug contents in Table 1). Cell viability was expressed as percentage *versus* control treatment and as an average ± S.D. of eight wells.

Anticancer activity of DOX-incorporated polymeric micelles was assessed with CD44 positive HepG2 cells and CD44 negative CT26 cells. As shown in Figure 8, DOX itself showed intrinsic cytotoxic properties both HepG2 and CT26 cells with dose-dependent manner and its cytotoxicity was not affected by HA pretreatment. At DOX PM treatment, viability of CT26 cells was not significantly affected by CD44 receptor blocking, indicating that endocytosis of HAgLG polymeric micelles did not enter by the receptor-mediated pathway. Interestingly, CD44 receptor blocking by pretreatment of HA significantly reduced anticancer activity of DOX-incorporated polymeric micelles while cell viability was not significantly changed in the absence of HA pretreatment compared to DOX treatment. These results indicated that HAgLG polymeric micelles have CD44 receptor specificity and CD44 receptor mediated anticancer activity. DOX itself was not affected by CD44 blocking at CD44-positive and negative cells. Furthermore, anticancer activity of DOX HAgLG polymeric micelles was not affected by CD44 receptor blocking at CD44-negative cells.

In conclusion, HAgLG polymeric micelles have small diameters less than 200 nm and are of spherical shape. They accelerated DOX release by hyaluronidase treatment. Furthermore, they have CD44 receptor specificity against HepG2 cells and CD44-receptor mediated anticancer activity against HepG2 cells.

## 3. Experimental Section

### 3.1. Materials

HA (molecular weight (*M*_W_) from manufacturer’s data: 8300 g/mol) was purchased from Lifecore Biomedical (Chaska, MN, USA). Triethylamine (TEA), phosphotungstic acid, sodium cyanoborohydride, hexamethylene diamine (HMDA), and fluorescein isothiocyanate (FITC) were purchased from Sigma Chem. Co. (St. Louis, MO, USA). *N*-(3-Dimethylaminopropyl)-*N*'-ethylcarbodiimide hydrochloride (EDAC) and *N*-hydroxysuccimide (NHS) were purchased from Aldrich Chemical Co. (St. Louis, MO, USA). The dialysis membranes (*M*_W_ cutoffs (MWCO): 12,000 g/mol) were purchased from Spectra/Pro™ Membranes. Dichloromethane, methanol and dimethyl sulfoxide (DMSO) were of HPLC grade or extra-pure grade. PCL PLGA-5005) were purchased from Wako Pure Chemicals Co. (Osaka, Japan). The weight and number average *M*_w_ of PLGA were 4920 and 4780 (polydispersity: 1.029), respectively, as reported previously [3].

### 3.2. Synthesis of HAgLG Copolymer

To synthesize amine-terminated PLGA, PLGA was activated with EDAC/NHS and then HMDA was added to the reaction as reported previously [3]. PLGA-amine dissolved in DMSO was reacted with HA (80 mg in 10 mL of H_2_O/DMSO mixtures (volume ratio: 2/8) in the presence of EDAC/NHS. After that, this solution was reacted for 3 days at room temperature. Resultant solution was precipitated into excess amount of methanol and filtered. Precipitates were washed with methanol and then white solid was dried in vacuo for 2 days. Solid was precipitated in the dichloromethane to remove unreacted PLGA and then precipitates were dried. Resultant products were stored in the refregarator.

### 3.3. Preparation of Polymeric Micelles

Empty polymeric micelles: 40 mg of HAgLG dissolved in 5 mL of H_2_O/DMSO (1/4, *v*/*v*) was poured into 15 mL of deionized water over 10 min to form micelles. The solvent was removed by dialysis against deionized water using a dialysis membrane (12,000 g/mol) for at least 1 day. Deionized water was exchanged at 1–2 h intervals during the dialysis procedure. Subsequently, the resulting solution was used for analysis or lyophilized.

DOX-incorporated polymeric micelles: 40 mg of HAgLG copolymer were dissolved in 5 mL of H_2_O/DMSO (1/4, *v*/*v*). DOX was dissolved in DMSO with trace amount of TEA and then added to HA solution. This solution was poured into 10 mL deionized water to form DOX-incorporated polymeric micelles and then dialyzed against deionized water using a dialysis membrane (12,000 g/mol) for 1 day. Water was exchanged 2 h intervals to remove solvent. Subsequently, the resulting solution was analyzed or lyophilized for 2 days (freeze dryer: FreeZone 1, Labconco Co., Kansas, MO, USA).

To measure DOX concentration in the polymeric micelles, 5 mg DOX-incorporated polymeric micelles were reconstituted in 1 mL deionized water and diluted with 9 mL DMSO. DOX concentration was evaluated using a UV-spectrophotometer (UV spectrophotometer 1201, Shimadzu Co., Tokyo, Japan) at 479 nm. Empty micelles were used as the blank. Drug content (%) = (drug weight in the polymeric micelles/total weight of polymeric micelles) × 100%.

Drug release study: 5 mg DOX-incorporated polymeric micelles were distributed into 5 mL phosphate buffered saline (PBS, 0.1 M, pH 7.4). This solution was introduced into dialysis membrane and then dialysis membrane was introduced into 50 mL tube with 45 mL of PBS. At specific time intervals, PBS was taken to measure released DOX and media was replaced with fresh PBS. DOX was measured with UV-spectrophotometer (UV spectrophotometer 1201, Shimadzu Co.) at 479 nm. To study the effect of hyaluronidase, 100 units of hyaluronidase was added to each dialysis membrane.

### 3.4. Analysis of HAgLG Polymeric Micelles

Characterization of the polymeric micelles was performed in DMSO or D_2_O by 500 MHz ^1^H NMR spectroscopy (500 MHz Superconducting FT-NMR Spectrometer, Unity-Inova 500). The morphology of micelles was observed using a transmission electron microscope (TEM, JEOL JEM-2000 FX II, Tokyo, Japan). The size of the polymeric micelles was measured with dynamic laser scattering (DLS-7000, Otsuka Electonics Co., Osaka, Japan).

### 3.5. Fluorescein Isothiocyanate (FITC)-Labelling of HAgLG

HAgLG-2 copolymer (100 mg) was dissolved in 10 mL H_2_O/DMSO (1/4, *v*/*v*) with 10 mg of FITC. This mixture was stirred for 3 h and then introduced into dialysis tube following dialysis against deionized water. Fluorescence intensity of water was checked using fluorescence spectroscopy (Shimadzu RF 5301 PC spectrofluorometer, Shimadzu Co.) and dialysis procedure was continued until no fluorescence could be measured.

### 3.6. Western Blot Analysis

For Western blotting, human hepatocellular carcinoma cell lines (SK Hep01 and HepG2), mouse colorectal carcinoma cell line (CT26 cell), oral squamous cell line (KB cell) were purchased from Korean Cell Line Bank (Seoul, Korea). KB cells and CT26 cells were cultured in RPMI1640 media supplemented with 10% FBS at 37 °C and 5% CO_2_. SK Hep01 cells and HepG2 cells were cultured in DMEM supplemented with 10% FBS at 37 °C and 5% CO_2_. Cells were harvested and lysed in a lysis buffer. The protein concentrations were determined using a protein assay kit (Bio-Rad, Hercules, CA, USA). Then, 50 µg of protein from the whole cell lysates was separated by 12% sodium dodecyl sulphate polyacrylamide gel electrophoresis and transferred to a polyvinylidene difluoride membrane (Pall Corporation, Port Washington, NY, USA**)**. Subsequently, the membrane was incubated for 2 h in TBS-T (10 mM TrisCl [pH 8.0], 150 mM NaCl, and 0.05% Tween 20) supplemented with 5% non-fat dry milk, and probed overnight at 4 °C with anti-human-CD44 antibody 1 (1:1000 dilution). The bound antibodies were visualized with an anti-human second antibody (1:20,000 dilution) conjugated with horseradish peroxidase using enhanced chemiluminescence reagents (Amersham Biosciences, Sunnyvale, CA, USA).

### 3.7. Receptor-Mediated Endocytosis of Polymeric Micelles into HepG2 Cells

To observe targetability of HAgLG polymeric micelles, HepG2 cells were employed. One hour before the addition of polymeric micelles, HA receptor (CD44) of tumor cells was blocked with 10 equivalents of HA *versus* polymeric micelles. Following this, FITC-labeled polymeric micelles were added and incubated for 1 h in a CO_2_ incubator. Cells were treated with 4% paraformaldehyde and fixed with immobilization solution (IMMU-MOUNT, Thermo Electron Corporation, Pittsburgh, PA, USA). Cells were observed with a confocal laser scanning microscope (CLSM, TCS-SP2; Leica, Wetzlar, Germany).

For flow cytometric analysis, cells were seeded in 6-well plates at a density of 1 × 10^6^ cells/well. Before the addition of polymeric micelles, CD44 receptor was blocked with 10 equivalents of HA. Following this, polymeric micelles were added to the cancer cells and incubated for 1 h. Cells were analyzed with flow cytometer (FACScan, Becton Dickinson, San Jose, CA, USA).

### 3.8. Cancer Cell Cytotoxicity

Hep G2 cells and CT26 cells were maintained in DMEM and RPMI1640 (10% fetal bovine serum, 1% antibiotics) at 5% CO_2_ incubator (37 °C). Anticancer activity of free DOX or DOX-incorporated polymeric micelles were evaluated with Hep G2 cells and CT26 cells by MTT proliferation assay. Then, 3 × 10^4^ cells were seeded in 96-well plates and incubated overnight with 5% CO_2_ incubator at 37 °C. DOX-incorporated micelles were exposed to these cells for 6 h. Following this, supernatent was discarded and replaced with fresh media. These cultures were further incubated for 24 h. After, 30 μL of MTT (5 mg/mL) was added to the 96-well plates and, 4 h later, 0.1 mL DMSO was added. The cell viability was evaluated by measurement of absorbance (560 nm test/630 nm reference) using microplate reader (Molecular Device Company, Sunnyvale, CA, USA).

### 3.9. Statistical Analysis

The results are expressed as the means of three parallel experiments ± standard deviation (S.D.). Statistical analysis of the results was performed using the *t* test with *p* < 0.05 as the minimal level of significance. For statistical analysis, SigmaPlot program (version 11.2, Systat software, Inc., San Jose, CA, USA) was used.

## 4. Conclusions

Self-assembled polymeric micelles of HAgLG graft copolymer was prepared for antitumor drug targeting against CD44 receptor of tumor cells. Polymeric micelles of HAgLG have spherical shapes and small particle sizes less than 100 nm. At ^1^H NMR study using D_2_O, the core-shell structure of HaLG polymeric micelles was confirmed. To confirm potential of receptor-mediated drug targeting, CD44-overexpressed HepG2 cells were employed. HepG2 cells treated with FITC-labeled HAbLG nanoparticles strongly expressed green fluorescence whereas fluorescence intensities of cells were significantly decreased by blocking of CD44 receptor. These results indicated that polymeric micelles can be used as targeting vehicles for CD44 receptor of tumor cells. Furthermore, the CD44-specific anticancer activity of HAgLG polymeric micelles was confirmed using CD44-negative CT26 cells and CD44-positive HepG2 cells. We suggest HAgLG polymeric micelles as a promising candidate for specific drug targeting.
